# Prevalence and knowledge of heavy menstrual bleeding among gynecology outpatients by scanning a WeChat QR Code

**DOI:** 10.1371/journal.pone.0229123

**Published:** 2020-04-02

**Authors:** Sisi Su, Xin Yang, Qing Su, Yang Zhao

**Affiliations:** 1 Department of Obstetrics and Gynecology, Peking University People’s Hospital, Beijing, China; 2 Department of Obstetrics and Gynecology, Fujian Nan’an Hospital, Fujian, China; Central South University, The Third Xiang Ya Hospital, CHINA

## Abstract

The aim of this study was to assess menstrual blood loss (MBL) and knowledge of heavy menstrual bleeding (HMB) among the gynecology outpatients at Peking University People’s Hospital, by scanning a WeChat (a social media application software developed by Tencent) QR Code using a mobile phone or tablet. This survey was conducted among outpatients who were treated at the Gynecology Department of Peking University People’s Hospital between September 2016 and November 2016. All participants filled up the questionnaires and scales through WeChat: general information questionnaire, Pictorial Blood Loss Assessment Chart (PBAC), Menorrhagia Multi-Attribute Quality-of-Life Scale (MMAS), and HMB knowledge questionnaire. Menstrual blood volume was assessed by the PBAC and self-assessment. Among the 1152 patients who filled out the survey, 77.4% (892/1152) had normal menstrual cycle (assessed by the patients), 15.6% (180/1152) patients described themselves as HMB, whereas the results from PBAC showed that 58.0% (668/1152) had HMB (PBAC ≥100). Among patients with PBAC ≥100, only 26.8% (179/668) patients reported HMB through self-assessment. Regarding its impact on daily life, the MMAS scores of HMB patients (PBAC ≥100) were significantly lower compared to those with normal blood loss (P<0.001). Regarding the awareness to HMB, 63.2% (728/1152) of the patients were not familiar with HMB. HMB is a common abnormal uterine bleeding and is frequently found among Chinese gynecology outpatients. HMB has major impacts on a woman’s quality of life, affecting both physical and emotional health domains. Since women generally have low levels of awareness and understanding of HMB, they could assess their blood loss using the PBAC, which they can forward to their health care provider using a mobile phone or tablet and the WeChat platform. This tool may be effortlessly used by the health care providers and patients to easily share HMB-related data.

## Introduction

Heavy menstrual bleeding (HMB), or excessive menstrual blood loss, can seriously affect the quality of life of women since it interferes with the physical, emotional, social, and material quality of life. HMB can occur alone or in combination with other symptoms [1]. It is a specific type of abnormal uterine bleeding (AUB) that may result from structural and non-structural abnormalities defined within the PALM-COEIN classification [2]. Most women with HMB do not have any structural or histologically identifiable abnormalities. HMB management is justified when HMB has an impact on quality of life. Menstrual blood loss (MBL) of >80 mL has traditionally been used as the criterion for HMB [3] along with the menstrual period lasting >7 days. These symptoms may occur either individually or together with other symptoms. Long-term HMB may lead to anemia, which in turn may cause a series of unfavorable consequences such as shortness of breath, tiredness, weakness, emotional fluctuation, and impaired knowledge [4]. In the United States, about 45% of the women have had a hysterectomy was because of menorrhagia [5]. The prevalence of HMB depends on the assessment, clinical setting, and cultural and social ideas of ‘normal’ menstruation [6,7]. Investigation and management are hampered by confusing and inconsistent nomenclature and the lack of a standardized investigation approach [2,3,7]. There is a low level of awareness and understanding of HMB amongst women, which often leads to acceptance of the condition, without seeking the medical help [7].

Currently, there are no data in China that could help us understand how often women suffer from HMB and how many women know that HMB could severely jeopardize their health. The physical assessment of MBL is difficult to achieve outside clinical research settings [1,3,8]. For healthcare professionals, volume and accuracy of data is impeded by confusing and inconsistent nomenclature [3,7], difficulty with traditional approaches to measurement of MBL [3,6,7], lack of a standardized investigation approach [2,7], and poor awareness and understanding of the impact of HMB amongst women [9,10]. Hence, the aim of this study was to determine MBL using a mobile phone or tablet to scan a WeChat (a social media application software developed by Tencent) QR Code in order to enter the mobile questionnaire survey system, and to examine the level of awareness and knowledge of HMB among the gynecology outpatients treated at Peking University People’s Hospital.

## Materials and methods

### Participants

This cross-sectional study included all consecutive outpatients who visited the Peking University People’s Hospital from September 2016 to November 2016 for various gynecological diseases or for a routine medical examination.

The inclusion criteria were: 1) an intact uterus; 2) with at least one ovary; 3) having menstruation; and 4) keeping a period diary or capable of recalling their menstruation-related facts. The exclusion criteria were: 1) pregnant women; 2) preadolescent or postmenopausal bleeding; 3) hormone replacement therapy due to primary amenorrhea or premature ovarian failure; 4) uterus/ovary removed on both sides; 5) use of steroid hormones in the past 3 months due to AUB; or 6) levonorgestrel-releasing intrauterine system (such as levonorgestrel intrauterine device) or implanted subdermal contraceptive. AUB was diagnosed based on the FIGO criteria [11].

### Assessment tools

The patients used a mobile phone or tablet to scan a WeChat QR Code to access the mobile questionnaire survey system. The study complied with the terms of service for the WeChat social media application software. All participants filled up the questionnaires and scales: general information questionnaire, period diary questionnaire, Menorrhagia Multi-Attribute Quality-of-Life Scale (MMAS), and menorrhagia knowledge questionnaire. The investigators were responsible for monitoring the results, and the physicians received training on the functions of the questionnaire system, the items included in the questionnaire, and the meaning of each item for implementing the survey.

### General information questionnaire

This questionnaire included items related to reasons for visiting the hospital, age, gestational history, menstrual cycle, self-assessment of menstrual blood volume, history of gynecological diseases (leiomyoma, adenomyosis, endometrial polyp, and endometrial hyperplasia), and history of gynecological surgery.

### Menstrual blood volume assessment

The patients were requested to use the Pictorial Blood Loss Assessment Chart (PBAC), a self-administered pictorial assessment chart that allocates scores reflecting the degree of staining of tampons and cotton-based sanitary pads during a menstrual cycle [8,12,13], to assess their menstrual blood volume, and upload the information by WeChat for 2 months. The PBAC has been validated [12,13]. They were instructed by their physicians regarding the use of the PBAC. PBAC scoring ≥100 for 2 months was considered as HMB, PBAC <10 was considered as hypomenorrhea, and PBAC scoring 10–99 was considered as normal menstrual flow [13]. All the sanitary products used were cotton-based. The PBAC score was automatically calculated by the mobile questionnaire survey system. Patients with hemoglobin (Hb) <115 g/L were diagnosed with anemia.

### MMAS

The MMAS [14,15] was used to measure the impact of HMB on the daily life of patients during menstruation. The MMAS includes six dimensions: practical difficulties, social life, family life, work and daily routine, psychological well-being, and physical health. Each patient was asked to construct a series of statements for each domain, and the final score was obtained based on the weighted score for each dimension and the weighted score for severity level. The score ranged from 0 (all dimensions in the worst state) to 100 (all dimensions in the best possible state). It was considered that daily life was affected when MMAS reached <100.

### HMB knowledge questionnaire

The HMB knowledge questionnaire was designed by the study authors and included three questions (S1 Material). (1) How much do you know about HMB: no knowledge, limited knowledge, partial knowledge, moderate knowledge, or adequate knowledge. (2) Which of the following do you agree with (multiple choices): profuse menstruation is a kind of disease that requires medical treatment; scanty menstruation is a kind of disease that requires medical treatment; consistent profuse menstruation without any other disease requires no medical treatment; consistent profuse menstruation without affecting the quality-of-life requires no medical treatment; consistent scanty menstruation with (without) any other disease requires no medical treatment; and consistent scanty menstruation without affecting the quality-of-life requires no medical treatment. (3) Do you believe that HMB is related to anemia: yes, no, or don’t know. If patients did not answer a question, the question was not included in the statistical analysis. This questionnaire was not validated.

### Statistical analysis

SPSS 17.0 (SPSS Inc., Chicago, IL) was used to perform the statistical analyses of the questionnaire data. Continuous data were expressed as means ± standard deviations (SD) and the differences between groups were analyzed using the Student’s t-test. Categorical data were expressed as frequency (percentage) and analyzed using the chi-square test. P-value <0.05 was considered statistically significant.

### Ethical approval

This study was approved by the ethics committee of Peking University People’s Hospital (No. 2015PHB087-01). All procedures were in accordance with the ethical standards of the institutional and national research committees, and in line with the 1964 Helsinki declaration and its later amendments or with comparable ethical standards. Informed consent was obtained from all participants included in the study. Because this survey did not involve interventions or supplementary medical procedures/testing, the patients provided their consent verbally to their physician. Then, completing the questionnaires was considered as a *de facto* consent. The ethics committee approved this consent procedure.

## Results

### Baseline characteristics

Among a total of 1736 patients who were invited to participate in the questionnaire survey, 584 patients were excluded from the study: 352 patients were excluded because they refused to fill the questionnaire and 232 patients were excluded because they had incomplete data. In total, 1152 patients completed the survey and were analyzed. The percentage of patients with normal menstrual cycle (assessed by the patients) was 77.4% (892/1152) (Table 1). The mean number of pregnancies was 1±1 (range, 0–7). The mean number of childbirth was 1±1 (range, 0–3). There was no patient taking oral contraceptive.

**Table 1:** Baseline characteristics of patients.

Variable	Subjects (n = 1152)
Age (years), n (%)	
≤30	370 (32.1)
31–40	450 (39.1)
>40	332 (28.8)
BMI (kg/m^2^), n (%)	
<19	63 (5.5)
19–24	692 (60.1)
>24	397 (34.5)
Gravidity, mean ± SD (range)	1±1 (0–7)
Parity, mean ± SD (range)	1±1 (0–3)
Normal menstrual cycle*, n (%)	892 (77.4)
Reasons of visit, n (%)	
N	739
Reproductive tract infection	267 (36.1)
Abnormal uterine bleeding	184 (24.9)
Gynecological tumor	106 (14.3)
Endocrine disease	30 (4.1)
Pelvic floor dysfunction	25 (3.4)
Intrauterine echogenic mass	20 (2.7)
Cervical intraepithelial neoplasia	12 (1.6)
Physical examination	56 (7.6)
Others	39 (5.3)

Normal menstrual cycle was assessed by the patients. BMI, body mass index; SD, standard deviation.

In total, 739 patients described their reasons for visiting the hospital. The major reason for visit was reproductive tract infection (36.1%, 267/739) including pelvic inflammation, cervicitis, cervical polyp, vaginitis, and vulvitis. The percentage of AUB (including all kinds of HMB) was 24.9% (184/739), the percentage of gynecological tumor (including ovary tumor, leiomyoma, endometrial cyst, adenomyosis, mesosalpinx cyst) was 14.3% (106/739) (Table 1).

### Self- and PBAC-based assessment of menstrual blood volume

**Fig 1:**
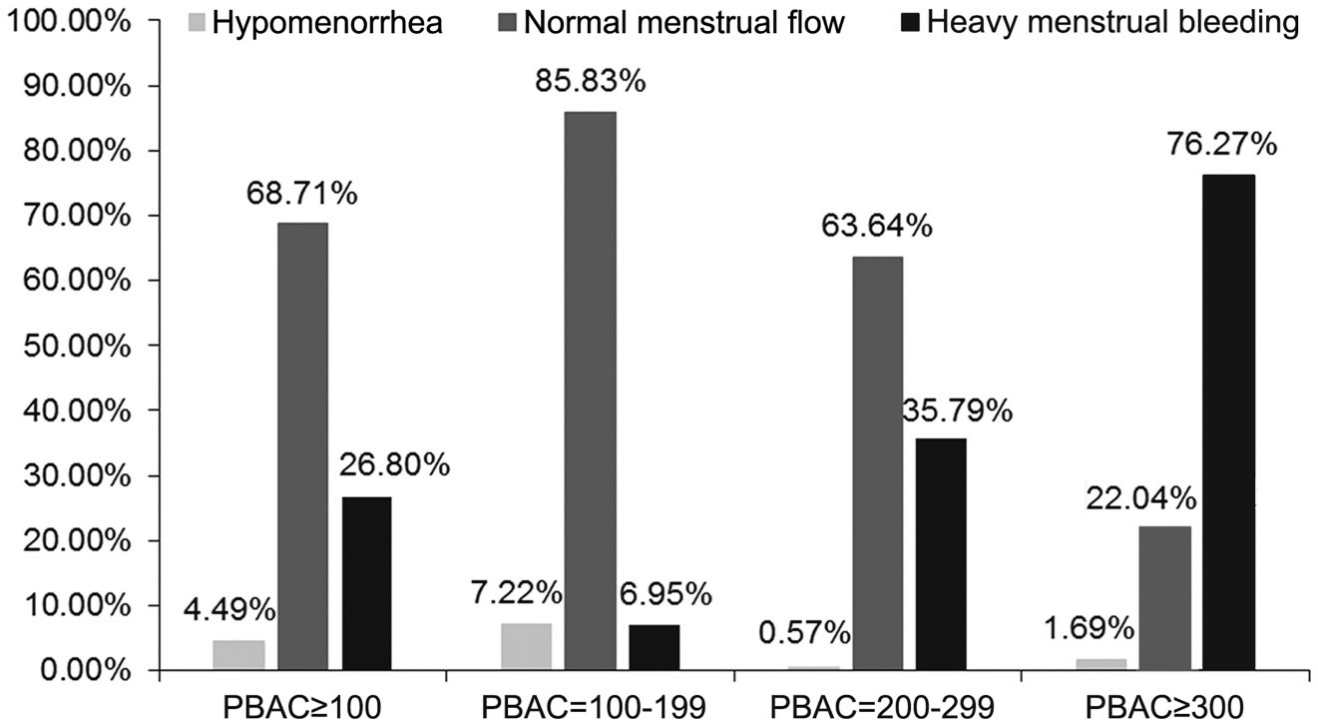
Pictorial blood loss assessment chart (PBAC)-based and self-assessment of menstrual blood volume in patients.

A total of 1152 patients assessed their own menstrual flow volume. The patient self-assessment of menstrual blood volume showed that HMB accounted for 15.6% (180/1152). After assessing menstrual blood volume using the PBAC, the percentage of PBAC ≥100 was 58.0% (668/1152) (Table 2). Among patients with PBAC ≥100, only 26.8% (179/668) reported HMB through self-assessment, and 7.0% (26/374), 35.8% (63/176), and 76.3% (90/118) patients self-reported as HMB among patients with PBAC 100–199, 200–299 and ≥300, respectively (Fig 1). Table 3 presents the PBAC scores of patients with according to the self-assessed menstrual blood volume.

**Table 2:** Proportion and MMAS total score of patients with different volume of MBL by self-assessment and PBAC assessment.

Variable	Self-assessment (n = 1152)	PBAC (n = 1152)	P
MBL, n (%)			<0.001
Hypomenorrhea	194 (16.8)	20 (1.7)	
Normal	778 (67.5)	464 (40.3)	
HMB	180 (15.6)	668 (58.0)	
MMAS total score, mean±SD			
Hypomenorrhea	94.4±10.3	92.8±6.0	<0.001
Normal	95.0±10.0	95.0±8.4	<0.001
HMB	73.4±24.1	89.2±18.6	<0.001

HMB, heavy menstrual bleeding; MBL, menstrual blood loss; PBAC, Pictorial Blood Loss Assessment Chart; SD, standard deviation.

**Table 3:** PBAC score of patients (n = 668) with different self-assessed menstrual blood volume and PBAC score of ≥100.

PBAC	Hypomenorrhea		Normal		HMB	
	n	Mean±SD	n	Mean±SD	n	Mean±SD
≥100	30	147.6±69.4	459	183.1±101.6	179	391.3±270.7
100–199	27	125.6±16.3	321	143.2±28.1	26	157.1±26.0
200–299	1	287	112	236.5±24.6	63	248.0±28.1
≥300	2	376.3±21.5	26	445.4±233.9	90	559.2±301.0

HMB, heavy menstrual bleeding; PBAC, Pictorial Blood Loss Assessment Chart; SD, standard deviation.

### AUB classification

In total, 60.8% (449/739) patients were diagnosed with AUB. According to the PBAC scores, 422 patients suffered from HMB, accounting for 94.0% of all AUB patients; 121 patients had a period >7 days, accounting for 26.9% of all AUB patients (121 patients had both PBAC ≥100 and a period >7 days).

### Quality-of-life of patients with HMB

The MMAS total scores are shown in Table 2. There was significant difference in MMAS total score among patients with different volume of MBL by self-assessment or PBAC assessment (P<0.001). A total of 152 patients who provided blood routine examination results were divided into two groups based on the PBAC score; 96 patients who scored PBAC ≥100 were assigned to the HMB group and 56 patients with PBAC <100 to the normal blood loss group. Regarding its impact on daily life, the MMAS scores of HMB patients were significantly lower compared to those with normal blood loss (P<0.001, Table 4). Among these patients, 64.6% (62/96) of patients from the HMB group suffered from anemia, compared to 3.6% (2/56) of patients from the normal blood loss group (P<0.001) (Table 4).

**Table 4:** The MMAS scores and hemoglobin levels of patients who provided the blood routine examination results.

Variable	PBAC ≥100 (n = 96)	PBAC <100 (n = 56)	P
MMAS, mean±SD			
Practical difficulties	8.3±4.7	13.5±1.4	<0.001
Social life	7.1±2.9	10±0	<0.001
Family life	19.7±6.5	23±0	<0.001
Physical health	13.8±7.6	21±0	<0.001
Work and daily routine	13.2±5.6	18±0	<0.001
Psychological well-being	10.3±4.3	14±0	<0.001
Total score	72.2±25.4	99.5±1.5	<0.001
Hemoglobin (g/L), mean±SD	105.5±22.0	129.2±17.3	<0.001
Anemia, n (%)	62 (64.6)	2 (3.6)	<0.001

MMAS, menorrhagia multi-attribute quality-of-life scale; PBAC, Pictorial Blood Loss Assessment Chart; SD, standard deviation.

### HMB knowledge of patients

According to the HMB knowledge questionnaire, 63.2% (728/1152) of the patients knew nothing about HMB, while 34.5% (397/1152) had limited knowledge of HMB. With regard to the medical treatment (multiple choices), 47.2% (544/1152) of the patients believed that profuse menstruation is a kind of disease that requires medical treatment, 48.2% (555/1152) believed that scanty menstruation is a kind of disease that requires medical treatment, 42.0% (484/1152) believed that consistent profuse menstruation without any other disease or without affecting the quality-of-life requires no medical treatment, and 18.1% (208/1152) believed that consistent scanty menstruation without any other disease or without affecting the quality-of-life requires no medical treatment. Regarding the connection between HMB and anemia, only 45.5% (524/1152) patients believed that the two are related, while 54.5% (628/1152) believed that there was no connection between the two or had no idea at all (Table 5).

**Table 5:** HMB knowledge of patients.

Item, n (%)	Subjects (n = 1152)
How much do you know about HMB	
No knowledge	728 (63.2)
Limited knowledge	397 (34.5)
Partial knowledge	16 (1.4)
Moderate knowledge	8 (0.7)
Adequate knowledge.	3 (0.3)
Which of the following do you agree with (multiple choices)	
Profuse menstruation is a kind of disease which requires medical treatment	544 (47.2)
Scanty menstruation is a kind of disease which requires medical treatment	555 (48.2)
Consistent profuse menstruation without any other disease or without affecting the quality-of-life requires no medical treatment	484 (42.0)
Consistent scanty menstruation without any other disease or without affecting the quality-of-life requires no medical treatment	208 (18.1)
HMB and anemia are related	524 (45.5)

HMB, heavy menstrual bleeding.

## Discussion

It is estimated that one in three women is affected by HMB [16,17], but the prevalence vary according to how HMB is assessed. Subjective or self-report measurement of HMB includes the overall impact on quality of life, which tends to result in higher prevalence compared with objective assessment [18]. A review of self-reported data (but based on data collected from the late ‘70s to early ‘90s) suggests that 8%-27% of women in developing countries are affected by HMB [19].

The physical objective quantitative assessment of MBL is difficult to achieve outside clinical research setting [3,8,20] where it can be measured by PBAC or alkaline haematin methods. In the present study, the patients were asked to assess their blood loss with the PBAC, and then used WeChat on their mobile phone or tablet to forward the data to the hospital. This tool was very easy to use. The patients could fill the mobile questionnaires at any time or anywhere.

In the present study, the PBAC scores demonstrated that 58.0% of patients suffered from HMB, but according to the self-assessment, only 15.6% patients were aware of it. Among patients who scored >100 on the PBAC, 68.7% patients described their MBL volume as normal. Even among patients with profuse menstruation (PBAC ≥300), some patients still described their MBL as normal or scanty. These differences might result from the traditional attitude towards menstruation and limited understanding of HMB. With increasing MBL, the proportion of women with Hb levels below the WHO threshold for anemia dramatically increased. According to Nelson et al. [21], HMB can lead to severe anemia (with Hb <5 g/dl). Janssen et al. (n = 313) found that anemia levels increase from 1.5% at an MBL of <20 ml, to 10.3% for an MBL between 61 and 80 ml, and to 50% for an MBL between 161 and 240 ml [22]. Another study in Chinese women (n = 421) revealed a similar relationship between Hb levels and the prevalence of HMB. At MBL <20 ml (n = 48), the prevalence was 0%; at MBL of 60–80 ml (n = 53), the prevalence was 17%; and at MBL>100 ml (n = 46), the prevalence was 26.1% [6]. The present study was an internet survey that assessed the prevalence and impact of HMB amongst women in the general population (aged 18–57 years). A total of 1225 women were identified as having experienced two or more of the predefined HMB symptoms over the past 12 months. The survey found that most women considered that HMB has a major negative impact on their lives, affecting their sexual life, physical activity, and productivity at work and at home [23].

Among our patients, 64.6% (62/96) from the HMB group and 3.6% (2/56) from the normal blood loss group suffered from anemia. Although HMB does not lead to critical diseases, chronic HMB causes iron-deficiency anemia that might have a strong impact on the daily life or physical/mental health of patients. According to the present study, the anemia rates among HMB patients appeared higher compared with patients with the normal MBL group. Regarding its impact on daily life, the MMAS scores of the subgroup of HMB patients who had routine laboratory examinations were significantly lower compared with those of the normal blood loss group, which subsequently had an impact on the quality-of-life in patients. This study showed that there is a low level of awareness and understanding of HMB amongst our patients. These findings are consistent with previous study that has shown that most women are not aware of HMB [24,25].

AUB is one of the common diseases encountered in gynecology clinics. In the present study, 60.8% of the patients were diagnosed with AUB, with HMB being the most common clinical manifestation (94.0%). Yet, the cause of HMB, which appears alone or with other symptoms, could lead to certain complications and thus should be investigated.

According to the present study, 63.2% of the patients had no knowledge of HMB. Based on verbal statements from the patients, they believe that the menstrual blood expels the toxins and contributes to good health, and that it is totally normal to have excessive menstrual bleeding. Unfortunately, this particular question was not included in the survey and we do not know the exact number of patients with this belief and the qualitative data of the conversations. Hence, 42.0% of the patients believed that consistent profuse menstruation without any other disease or negative effect on the quality of life does not require any medical treatment. Only 45.5% knew that HMB is closely related with anemia. In a questionnaire survey involving 6179 female patients aged 18–55 from 15 countries, 59% of the patients described the above-than-average menstrual bleeding as normal, 41% believed that there is no cure even if they go see a health care provider, and only 35% discussed the problem of HMB with a health care provider [7]. Our results revealed that very few patients had knowledge of HMB, while most believed that HMB requires no medical treatment. This is a common fact in China, as well as in other countries, which is why patients and health care providers should work in the education of women regarding HMB, thus improving the awareness of HMB, its possible harmful consequences and facilitating the search for medical attention in a timely manner, avoiding the appearance of complications. PBAC <100 was observed in a small part of patients with self-assessed HMB. These patients indeed had normal blood loss or hypomenorrhea. They paid more attention to HMB or they were engaged in professions related to medicine.

Even though it is true that a large proportion of the participants in the study were seeking medical work-up for AUB, there was still a very low level of awareness and understanding of HMB, a common manifestation of AUB, amongst these women. This suggests that even the women who are treated for AUB and are in direct contact with health care providers have a very low understanding of HMB. Consequently, the suggested approach could be used to overcome this limitation and lack of knowledge and understanding, where individuals, patients or not, could use their mobile phones or tablets to assess blood loss using the PBAC, which they could send to the health care providers via WeChat. This tool may help health care providers and patients easily communicate and to accurately ascertain whether the patient suffers from HMB. Another benefit is that the patients might feel more at ease reporting their condition in such way, than trying to describe it given that this condition might yet be a sensitive topic, especially in less developed or rural areas.

There are some limitations in this study. First, although there were 1152 patients in the present study, the sample size might be small because sample size calculation was not performed. This was a single-center study of all patients who scanned the QR code, without formal or active sampling from our part, and the generalizability might be inadequate. Second, there might be a risk for bias from patients who decided not to participate in the study and from patients who did not provide the reasons for visiting hospital. Third, the original PBAC was developed as paper version, and we did not validate the difference when we transferred it from paper to electronic format. Different screen sizes might influence the judgment of women in blood loss, but this could be overcome in the future by adding some kind of metric to the charts. In addition, even if the PBAC is recognized as accurate, it is still a semi-quantitative method [12,13]. Fourth, the HMB knowledge questionnaire is not validated. Finally, the first question of the HMB questionnaire was a subjective cognitive question.

## Conclusion

HMB is frequently found among women consulting to a gynecological clinic, and is a common manifestation of AUB. Among the subgroup of patients who underwent routine laboratory tests, HMB affects a woman’s physical and emotional quality of life. There is a low level of awareness and understanding of HMB amongst women. We suggest an effective approach where patients can use their mobile phones or tablets to assess blood loss using PBAC, which they can send to hospital via WeChat. This tool may help health care providers and patients easily and accurately ascertain whether the patient suffers from HMB.

## Supporting information

S1 Material Heavy menstrual bleeding (HMB) knowledge questionnaire (English and Chinese version). (DOCX)

S1 Checklist (DOC)

S1 Data (ZIP)

## References

[1] NICE. Heavy menstrual bleeding: assessment and management. NICE clinical guidelines [CG44]. 2007. Last updated: August 2016. Accessed at nice.org.uk/guidance/cg44.

[2] Munro MG, Critchley HO, Broder MS, Fraser IS, Disorders FWGoM (2011) FIGO classification system (PALM-COEIN) for causes of abnormal uterine bleeding in nongravid women of reproductive age. Int J Gynaecol Obstet 113: 3–13. 10.1016/j.ijgo.2010.11.011 21345435

[3] Bahamondes L, Ali M (2015) Recent advances in managing and understanding menstrual disorders. F1000Prime Rep 7: 33 10.12703/P7-33 25926984 PMC4371378

[4] Bonafede MM, Miller JD, Laughlin-Tommaso SK, Lukes AS, Meyer NM, Lenhart GM (2014) Retrospective database analysis of clinical outcomes and costs for treatment of abnormal uterine bleeding among women enrolled in US Medicaid programs. Clinicoecon Outcomes Res 6: 423–429. 10.2147/CEOR.S67888 25336979 PMC4199837

[5] Cohen SL, Vitonis AF, Einarsson JI (2014) Updated hysterectomy surveillance and factors associated with minimally invasive hysterectomy. JSLS 18. 10.4293/JSLS.2014.00096 PMC4208898 25392662

[6] National Collaborating Centre for Women’s and Children’s Health (2007) Heavy Menstrual Bleeding Clinical Guideline 44. London: RCOG Press for NICE.

[7] Bitzer J, Serrani M, Lahav A (2013) Womens attitudes towards heavy menstrual bleeding and their impact on quality of life. Open Access Journal of Contraception 4: 21–28.

[8] The Menorrhagia Research Group (2004) Quantification of menstrual blood loss. The Obstetrician & Gynaecologist 6: 88–92.

[9] Munro MG (2017) Practical aspects of the two FIGO systems for management of abnormal uterine bleeding in the reproductive years. Best Pract Res Clin Obstet Gynaecol 40: 3–22. 10.1016/j.bpobgyn.2016.09.011 27836285

[10] Byles JE, Hanrahan PF, Schofield MJ (1997) 'It would be good to know you’re not alone’: the health care needs of women with menstrual symptoms. Fam Pract 14: 249–254. 10.1093/fampra/14.3.249 9201501

[11] Fraser IS, Critchley HO, Broder M, Munro MG (2011) The FIGO recommendations on terminologies and definitions for normal and abnormal uterine bleeding. Semin Reprod Med 29: 383–390. 10.1055/s-0031-1287662 22065325

[12] Zakherah MS, Sayed GH, El-Nashar SA, Shaaban MM (2011) Pictorial blood loss assessment chart in the evaluation of heavy menstrual bleeding: diagnostic accuracy compared to alkaline hematin. Gynecol Obstet Invest 71: 281–284. 10.1159/000320336 21228538

[13] Higham JM, O’Brien PM, Shaw RW (1990) Assessment of menstrual blood loss using a pictorial chart. Br J Obstet Gynaecol 97: 734–739. 10.1111/j.1471-0528.1990.tb16249.x 2400752

[14] Pattison H, Daniels JP, Kai J, Gupta JK (2011) The measurement properties of the menorrhagia multi-attribute quality-of-life scale: a psychometric analysis. BJOG 118: 1528–1531. 10.1111/j.1471-0528.2011.03057.x 21790952

[15] Shaw RW, Brickley MR, Evans L, Edwards MJ (1998) Perceptions of women on the impact of menorrhagia on their health using multi-attribute utility assessment. Br J Obstet Gynaecol 105: 1155–1159. 10.1111/j.1471-0528.1998.tb09968.x 9853763

[16] Hurskainen R, Grenman S, Komi I, Kujansuu E, Luoto R, Orrainen M, et al (2007) Diagnosis and treatment of menorrhagia. Acta Obstet Gynecol Scand 86: 749–757. 10.1080/00016340701415400 17520411

[17] Singh S, Best C, Dunn S, Leyland N, Wolfman WL, Clinical Practice GC (2013) Abnormal uterine bleeding in pre-menopausal women. J Obstet Gynaecol Can 35: 473–475. 10.1016/S1701-2163(15)30939-7 23756279

[18] Fraser I (2009) Health-related quality of life and economic burden of abnormal uterine bleeding. Expert Rev Obstet Gynecol 4: 179–189.

[19] Harlow SD, Campbell OM (2004) Epidemiology of menstrual disorders in developing countries: a systematic review. BJOG 111: 6–16. 10.1111/j.1471-0528.2004.00012.x 14687045

[20] Zander R, Lang W, Wolf HU (1984) Alkaline haematin D-575, a new tool for the determination of haemoglobin as an alternative to the cyanhaemiglobin method. I. Description of the method. Clin Chim Acta 136: 83–93. 10.1016/0009-8981(84)90250-x 6692568

[21] Nelson AL, Ritchie JJ (2015) Severe anemia from heavy menstrual bleeding requires heightened attention. Am J Obstet Gynecol 213: 97 e91-96. 25935784 10.1016/j.ajog.2015.04.023

[22] Janssen CA, Scholten PC, Heintz AP (1998) Reconsidering menorrhagia in gynecological practice. Is a 30-year-old definition still valid? Eur J Obstet Gynecol Reprod Biol 78: 69–72. 10.1016/s0301-2115(97)00275-3 9605452

[23] Fraser IS, Mansour D, Breymann C, Hoffman C, Mezzacasa A, Petraglia F (2015) Prevalence of heavy menstrual bleeding and experiences of affected women in a European patient survey. Int J Gynaecol Obstet 128: 196–200. 10.1016/j.ijgo.2014.09.027 25627706

[24] Hallberg L, Hogdahl AM, Nilsson L, Rybo G (1966) Menstrual blood loss—a population study. Variation at different ages and attempts to define normality. Acta Obstet Gynecol Scand 45: 320–351. 10.3109/00016346609158455 5922481

[25] Fraser IS, McCarron G, Markham R (1984) A preliminary study of factors influencing perception of menstrual blood loss volume. Am J Obstet Gynecol 149: 788–793. 10.1016/0002-9378(84)90123-6 6380294

