# Supplementary material for: Prevalence and knowledge of heavy menstrual bleeding among gynecology outpatients by scanning a WeChat QR Code
Source: PLoS One. 2020 Apr 2;15(4):e0229123. doi: 10.1371/journal.pone.0229123 (PMC7117654; doi:10.1371/journal.pone.0229123)
Supplement: S1 Material — (DOCX) [file pone.0229123.s001.docx]

**Heavy menstrual bleeding (HMB) knowledge questionnaire (English version)**

1. How much do you know about HMB:

a. no knowledge b. limited knowledge c. partial knowledge d. moderate knowledge e. adequate knowledge

2. Which of the following do you agree with (multiple choices):

a. Profuse menstruation is a kind of disease which requires medical treatment.

b. Scanty menstruation is a kind of disease which requires medical treatment.

c. Consistent profuse menstruation without any other disease requires no medical treatment.

d. Consistent profuse menstruation without affecting the quality-of-life requires no medical treatment

e. Consistent scanty menstruation without any other disease requires no medical treatment.

f. Consistent scanty menstruation without affecting the quality-of-life requires no medical treatment

3. The relationship between HMB and anemia:

a. related b. not related c. unknown

**Heavy menstrual bleeding (HMB) knowledge questionnaire (Chinese version)**

**月经过多知识问卷**

1.对月经过多的认识：

a完全不了解 b了解有限 c部分了解 d中度了解 e充分了解

2.你同意以下哪些说法（多选题）：

a经量多是一种病，需要去医院看病

b月经量少是一种病，需要去医院看病

c一贯的月经量多，如果没有其他疾病，无需看病

d一贯的月经量多，如果没有影响生活质量，无需看病

e一贯的月经量少，如果没有其他疾病，无需看病

f一贯的月经量少，如果没有影响生活质量，无需看病

3.对月经过多与贫血的关系：

a有关系b没有关系c不知道
